# Diagnostic certainty during in‐person and telehealth autism evaluations

**DOI:** 10.1002/jcv2.12201

**Published:** 2023-10-28

**Authors:** Natasha N. Ludwig, Calliope Holingue, Ji Su Hong, Luther G. Kalb, Danika Pfeiffer, Rachel Reetzke, Deepa Menon, Rebecca Landa

**Affiliations:** ^1^ Department of Neuropsychology Kennedy Krieger Institute Baltimore Maryland USA; ^2^ Department of Psychiatry and Behavioral Sciences Johns Hopkins School of Medicine Baltimore Maryland USA; ^3^ Center for Autism and Related Disorders Kennedy Krieger Institute Baltimore Maryland USA; ^4^ Department of Mental Health Johns Hopkins Bloomberg School of Public Health Baltimore Maryland USA; ^5^ Department of Neurology Johns Hopkins School of Medicine Baltimore Maryland United States

**Keywords:** ADHD, assessment, autism spectrum disorder, diagnosis, intellectual disability

## Abstract

**Background:**

Many diagnostic evaluations abruptly shifted to telehealth during the COVID‐19 pandemic; however, little is known about the impact on diagnosis patterns for children evaluated for autism spectrum disorder (ASD). The purpose of this clinical research study was to examine (1) the frequency of diagnoses evaluated beyond ASD; (2) the frequency of diagnoses made, including ASD; and (3) clinician diagnostic certainty for all diagnoses evaluated for children who received an evaluation due to primary concerns about ASD via telehealth during the pandemic compared to those evaluated in person before the pandemic at an ASD specialty clinic.

**Methods:**

The sample included 2192 children, 1–17 years (*M* = 6.5 years; *SD* = 3.9), evaluated by a physician/psychologist at an ASD specialty center. A total of 649 children were evaluated in‐person September 1, 2019–March 13, 2020 (pre‐pandemic) and 1543 were evaluated via telehealth March 14, 2020–July 26, 2021 (during pandemic). Upon completion of each evaluation, clinicians provided a final diagnostic determination (i.e., “*Yes,” “No,” “Possible,”* or “*Not Assessed”*) for the following DSM‐5 conditions: ASD, attention‐deficit/hyperactivity disorder (ADHD), intellectual developmental disorder (IDD), anxiety (ANX), depression (DEP), and behavioral disorder (BD). “*Possible*” indicated lower certainty and the diagnosis was not provided. “*Not Assessed*” indicated the disorder was not evaluated.

**Results:**

Diagnostic certainty for ASD and ADHD was lower and clinicians evaluated for and made diagnoses of IDD less often during evaluations that occurred via telehealth during the pandemic versus in person before the pandemic. DEP and BD were diagnosed more frequently, diagnostic certainty of DEP was lower, and no differences in the frequency of ANX diagnoses emerged during evaluations conducted via telehealth during the pandemic compared to those conducted in person before the pandemic.

**Conclusions:**

Differences emerged in the frequency of diagnoses evaluated and made and diagnostic certainty for evaluations conducted via telehealth during the pandemic compared to in person before the pandemic, which likely impacted patients and reflect real‐word challenges. Future work should examine whether these patterns are generalizable and the mechanisms that contribute to these differences.

## Graphical abstract

The purpose of this study was to examine (1) the frequency of diagnoses evaluated for beyond autism spectrum disorder (ASD); (2) the frequency of diagnoses actually made, including ASD; and (3) clinician diagnostic certainty for children who received an ASD evaluation via telehealth during the pandemic compared to those evaluated in person before the pandemic. Diagnostic certainty for ASD and attention‐deficit/hyperactivity disorder was lower and clinicians evaluated for and made diagnoses of intellectual developmental disorder less often during evaluations that occurred via telehealth versus in person. Depression (DEP) and behavioral disorder diagnoses were more frequent, diagnostic certainty of DEP was lower, and no differences were observed for anxiety when comparing evaluations conducted via telehealth to in person.

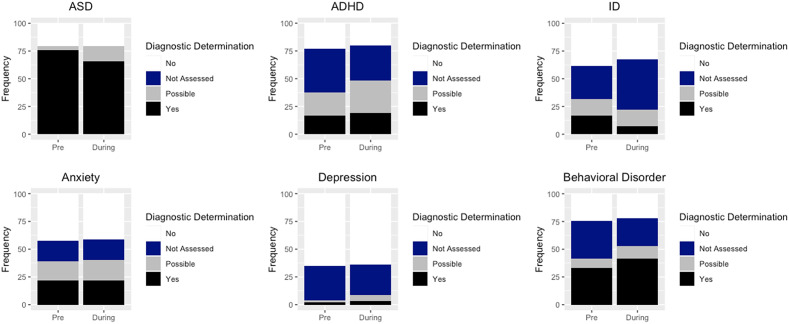

**Key points**

- This study demonstrated differences in diagnosis patterns and diagnostic certainty at our autism specialty center for clinical evaluations conducted in person before the pandemic compared to those conducted via telehealth during the pandemic.
- Results showed lower diagnostic certainty for autism spectrum disorder (ASD) and attention‐deficit/hyperactivity disorder and less frequent evaluation of and diagnoses of intellectual developmental disorder during ASD diagnostic evaluations that occurred via telehealth during the pandemic compared to those conducted in person before the pandemic. Additionally, diagnoses of depression and behavioral disorders were made more often during the pandemic.
- Future work is needed to examine generalizability of these findings to other clinical centers and the mechanisms that may be contributing to these differences.

## INTRODUCTION

Early and accurate diagnosis of childhood neurodevelopmental and psychiatric conditions is critical for prompt access to appropriate interventions; however, the process of making diagnostic decisions is complex. There is convention for conducting diagnostic evaluations for neurodevelopmental conditions such as autism spectrum disorder (ASD) within the context of in‐person evaluation (Hyman et al., 2020); however, research on efficacious, valid, and reliable methods of conducting evaluations to diagnose ASD and other neurodevelopmental disorders via telehealth is sparse. Two recent scoping reviews (Alfuraydan et al., 2020; Stavropoulos et al., 2022) revealed that pre‐pandemic diagnoses of ASD via telehealth were generally consistent with in‐person diagnostic evaluations. For example, Corona et al. (2021) found that ASD diagnoses made via telehealth were in agreement with an in‐person ASD diagnosis 86% of the time. There also is evidence that telehealth‐based evaluation of ASD is perceived to be feasible and acceptable by both clinicians and families (Corona et al., 2021; Gibbs et al., 2021; Matthews et al., 2021). Benefits of telehealth include increased patient access, the ability for providers to view behaviors in naturalistic environments, and inclusion of a broader range of family members in the evaluation process; however, much of the evidence for the efficacy of telehealth diagnostic evaluation of ASD has come from studies conducted in a controlled clinical laboratory setting (Alfuraydan et al., 2020). Before telehealth can be regarded as an effective modality for ASD diagnostic evaluation, additional large‐scale investigations conducted in community‐based clinical settings are needed.

Due to social distancing restrictions and the pause in in‐person clinical services with the onset of the COVID‐19 pandemic, clinicians across the world were required to rapidly develop new ways to meet patients' needs (see Berger et al., 2021; Dow et al., 2022; Jang et al., 2021; Ludwig et al., 2022; Wagner et al., 2021 for reviews of measures and clinical approaches). While there was a small, growing evidence‐base for telehealth prior to the pandemic, most ASD diagnosticians had minimal to no practical experience with telehealth evaluation (Kryszak et al., 2022). Recent evidence from a study including a small number of psychologists (*N* = 7) evaluating individuals from toddlerhood through adulthood for ASD suggests that they reported similar “confidence in conducting” the interview aspect of the evaluation (i.e., Autism Diagnostic Interview, Revised; Le Couter et al., 2003), but less confidence with observational aspects of the evaluation (i.e., Autism Diagnostic Observation Schedule, Second Edition ADOS; Lord et al., 2012) when conducted via telehealth during the pandemic compared to in person evaluations before the pandemic (Gibbs et al., 2021). It is important to note that telehealth administration of the ADOS is not valid so a clinical observation protocol using activities from the ADOS was administered when evaluations were conducted via telehealth in this study. This “ADOS‐informed assessment” approach became quite common across clinical centers during the pandemic (Spain et al., 2022). Since clinician “confidence in conducting” evaluations was lower via telehealth during the pandemic, it is reasonable to hypothesize that their confidence or certainty in making an ASD diagnosis may have also been lower; however, this is a very small sample of clinicians and research in larger samples is needed.

Prior to the COVID‐19 pandemic, McDonnell et al. (2019) demonstrated that clinicians (i.e., psychologists and developmental pediatricians) were “completely certain” of an in‐person ASD diagnosis 60% of the time in their sample of 478 toddlers at‐risk for ASD. Interestingly, these clinicians reported a higher frequency of diagnostic certainty when they provided an ASD diagnosis versus when they ruled it out (70.3% completely certain vs. 31.5% completely certain, respectively). Reduced certainty was also related to child factors including moderate ASD symptom presentation (compared to low or high symptoms), older child age, and higher cognitive and adaptive functioning. While this study sample presented variability in diagnostic certainty, further research is needed to better elucidate factors contributing to diagnostic certainty in larger samples, across a broader age range, and when evaluation is completed over telehealth.

Wagner et al. (2021) published initial findings exploring the utility of a telehealth‐to‐home model of ASD diagnostic evaluation for 204 toddlers during the pandemic. To our knowledge, this is the first published study examining diagnostic certainty of ASD during the pandemic via telehealth. Nine providers were trained on a telehealth‐administered assessment of ASD symptoms in toddlers, the TELE‐ASD‐PEDS (TAP; Corona et al., 2020). Using this assessment tool, providers were more certain when making a clinical diagnosis of ASD (*M* = 3.77, *SD* = 0.46; 1 = completely uncertain and 4 = completely certain) than when ruling it out (*M* = 2.83, *SD* = 0.72, *p* < 0.001). Furthermore, all providers reported being comfortable making a diagnosis of ASD in toddlers following a telehealth evaluation (i.e., 33% were *Very Comfortable*, 56% were *Comfortable*, and 11% were *Mildly Comfortable*). While these promising data are important for understanding the utility of the TAP for ASD evaluation and diagnosis in toddlers via telehealth, findings are limited to ASD evaluations using the TAP assessment tool and a relatively small sample.

It is important to acknowledge that children who present for an ASD evaluation, but do not meet diagnostic criteria, often have a complex neurodevelopmental presentation. This can be seen in children showing risk for ASD via the Modified Checklist for Autism in Toddlers, Revised with Follow Up (M‐CHAT‐R/F). In large population‐based studies, almost half of toddlers who screen positive, but do not meet ASD criteria, meet criteria for some other developmental delay or concern (e.g., global developmental delay, language disorder; Robins et al., 2014). It is also important to recognize that children with ASD often present with co‐occurring neurodevelopmental or psychiatric conditions that require prompt clinical attention (Mannion & Leader, 2016; Rosen et al., 2018). Taken together, community providers who conduct ASD differential diagnostic evaluations via telehealth must be equipped to assess for and diagnose a range of childhood conditions commonly observed in those referred for ASD concerns such as attention‐deficit/hyperactivity disorder (ADHD), global developmental delay/intellectual developmental disorder (IDD), anxiety (ANX), depression (DEP), and behavioral disorders (BD). This is of utmost importance within the context of the pandemic considering reports of higher rates of psychiatric conditions during this period (Vasa et al., 2021).

In general, psychiatric diagnoses (e.g., ANX, DEP) made via telehealth are highly consistent with in‐person services for both adult (O’Reilly et al., 2007) and child/adolescent populations (Elford et al., 2000); however, less is known about the evaluation of disorders conventionally relying on at least some degree of direct observation of behavior, such as the diagnoses designated in the Diagnostic and Statistical Manual of Mental Disorders, Fifth Edition (DSM‐5; American Psychiatric Association, 2013) as “neurodevelopmental conditions” (e.g., ADHD and IDD). Specifically, in diagnosing ANX, DEP, and BD in children, caregiver and child (if able) report of symptoms is often weighed heavily in diagnostic decision‐making, particularly because symptom presentation can vary significantly across contexts. In contrast, while report of behavior remains necessary in the diagnosis of neurodevelopmental disorders (e.g., ASD, ADHD, IDD), diagnosis is also dependent on clinician observation of behavior, due to the presumed pervasiveness of symptoms. This is especially the case for ASD and IDD, and perhaps to a lesser extent, ADHD. For example, standard of care in diagnosing ASD involves an objective observation of behavior (Hyman et al., 2020) and a diagnosis of IDD requires consideration of performance on standardized intellectual testing (American Psychiatric Association, 2013). While there is emerging research supporting concordance between in‐person and telehealth administered standardized intellectual testing (Hamner et al., 2021; Harder et al., 2020; McDermott et al., 2023; Ng et al., 2023; Wright, 2020), much less is known about frequency at which providers are evaluating for IDD over telehealth or their certainty in making the diagnosis within the context of this evaluation modality. Notably, there has been very little research comparing evaluation of neurodevelopmental and psychiatric disorders other than ASD before and during the pandemic.

Taken together, providers evaluating children due to concern for ASD must be able to assess for and diagnose a range of neurodevelopmental and psychiatric conditions during the pandemic to ensure patients' needs are met. Telehealth evaluation has promise for supporting the diagnostic process during the pandemic, but information about the utility of this modality is important. At present, no published research, to our knowledge, has addressed whether there were differences in diagnosis patterns and diagnostic certainty within the context of ASD evaluations conducted via telehealth during the pandemic compared to those conducted in person pre‐pandemic within a clinic setting. The purpose of this study was to address this dearth in evidence by examining: (1) the frequency of diagnoses beyond ASD that were evaluated (i.e., ADHD, IDD, ANX, DEP, and BD), (2) the number of diagnoses actually made, including ASD, and (3) the confidence providers had in making these diagnoses for evaluations conducted via telehealth during the pandemic compared to evaluations conducted in person pre‐pandemic for children presenting for clinical evaluation with a primary concern of ASD at our center. We hypothesized ASD would be diagnosed less often and that ASD diagnostic certainty would be lower for evaluations conducted via telehealth compared to in person. We based this hypothesis on the assumption that ASD is highly reliant on clinician observation of behavior and that ASD telehealth assessment protocols and clinicians' experience with telehealth assessment were in an early stage at the time of the study. For similar reasons, we hypothesized that other neurodevelopmental disorders (i.e., ADHD and IDD) would be evaluated and diagnosed less often and that diagnostic certainty would be lower when comparing evaluations conducted via telehealth to those conducted in person. Regarding the psychiatric diagnoses examined (i.e., ANX, DEP, BD), it was hypothesized these conditions would be evaluated and diagnosed more often via telehealth given the higher prevalence of these conditions during the pandemic (Vasa et al., 2021). We also posited that diagnostic certainty for these conditions would be equivalent across evaluation cohorts given previous work demonstrating consistency in services provided via telehealth and in‐person.

## METHODS

### Participants

The sample included 2192 children (Table 1), evaluated due to concerns about ASD at a large, university‐affiliated, ASD specialty center located in an urban setting within the Mid‐Atlantic region of the United States. The clinic provides medical and therapeutic services to children with ASD. Children may be referred by an internal provider (e.g., physician, psychologist, therapist from another department) or external provider (e.g., community‐based primary care, school, therapist, etc.). Patients may receive evaluation for initial diagnosis, second opinions, and/or diagnostic clarity of ASD and common co‐occurring conditions.

**TABLE 1:** Participant demographics and provider information.

	Total (*n* = 2192)	In‐person (*n* = 649)	Telehealth (*n* = 1543)	*p*‐value overall
Age (years; *M* (*SD*))	6.5 (3.9)	7 (3.9)	6.3 (3.8)	0.003
Categorical age (years; %)				
Less than 3	18.7	14.6	20.4	0.022
3–4	25.8	24.5	26.4	
5–6	18.1	18.6	17.8	
7–8	13.9	15.1	13.4	
9–10	9.1	10.6	8.5	
11–12	5.7	6.0	5.5	
13–14	4.7	5.2	4.5	
15–17	4.0	5.2	3.5	
Sex (%)				
Male	78.2	82.4	76.4	0.002
Female	21.8	17.6	23.6	
Race (%)				
White	41.9	46.5	40.0	<0.0001
Black/African‐American	30.4	29.0	31.0	
Asian	6.5	9.1	5.4	
Multiracial	5.8	2.0	7.5	
Hispanic	3.4	1.2	4.3	
Other	5.1	10.2	3.0	
Unknown	6.8	2.0	8.8	
Insurance (%)				
Commercial	50.1	58.6	46.5	<0.0001
Public	49.8	41.4	53.3	
Self‐pay	0.1	0.0	0.1	
Provider type (%)				
Physician	48.8	45.9	50.0	0.20
Psychologist	51.2	54.1	50.0	

**FIGURE 1:**
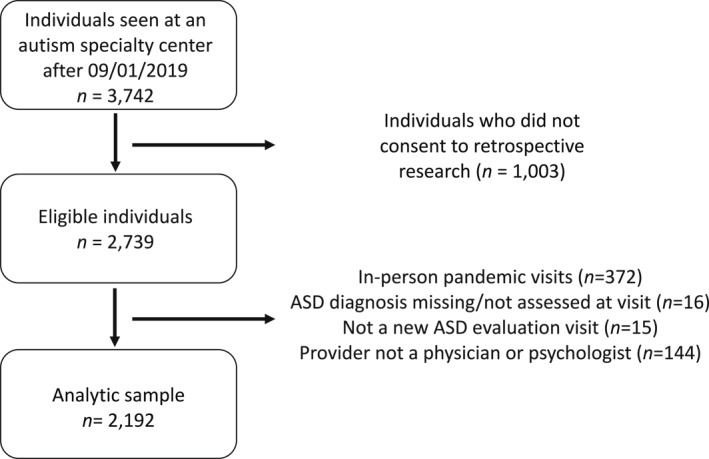
Sample derivation.

A total of 649 children who received an in‐person diagnostic evaluation between September 1, 2019 and March 13, 2020 (pre‐pandemic) and 1543 who received a telehealth diagnostic evaluation between March 14, 2020 and July 26, 2021 (during pandemic) were included in this study. Children's data were included if their parent/guardian consented to participate in the center's institutional review board‐approved clinical research registry (IRB NA_00010880). The consent rate for the registry is approximately 80% (Kalb et al., 2019). Those included also had the diagnostic variables of interest available in the electronic medical record (EMR; i.e., diagnostic classification table; DCT). Notably, children were excluded if they were evaluated via telehealth prior to the pandemic or if they were evaluated in‐person during the pandemic, both of which occurred infrequently at our center. Children were also excluded if they were not evaluated for an initial ASD diagnosis or if they were evaluated by a professional other than a licensed physician or psychologist within our ASD center (Figure 1).

Participants were between the ages of 1 and 17 years (*M* = 6.7, *SD* = 4.1) and were primarily male (78.2%). Compared to participants evaluated in person, the cohort evaluated via telehealth was slightly younger (*M* = 6.5 years, *SD* = 4.0 vs. *M* = 7.2 *SD* = 4.3) and included more females (i.e., 23.6%, vs. 17.6%). Notably, racial diversity was greater and there were more children with public insurance (41.4% vs. 53.3%, respectively with public insurance) in the cohort evaluated via telehealth compared to in person. The ratio of physicians versus psychologist serving as diagnostician remained consistent across evaluation cohorts (Table 1).

### Procedure

Patients were referred to specific types of clinicians and teams based on referral reason/question(s), age, and functioning level (see Ludwig et al., 2022 for more information about triaging at our ASD specialty clinic). Most ASD evaluations conducted at our center are team‐based. Diagnostic teams consisted of the diagnosing physician (e.g., psychiatrist, developmental pediatrician, neurologist) or psychologist in tandem with an occupational therapist, speech‐language pathologist (SLP), and/or social worker. Team evaluations typically took place on the same day or same week. Final clinical best‐estimate (CBE) diagnoses made during team evaluations were consensus‐based. Non‐team evaluations were conducted by a licensed physician or psychologist only who determined the final CBE diagnoses independently. After each diagnostic evaluation, the physician or psychologist was required to complete the DCT in the EMR when documenting the evaluation (*N* = 28 unique diagnosing providers were included in this study), which is the point of final diagnostic determination. If both a physician and psychologist were part of the diagnostic team, the DCT completed by the provider who saw the child first was used for the purposes of this study. All diagnoses were determined by DSM‐5 criteria (Table 2). The majority of diagnosing providers included in this study (*N* = 21) conducted both evaluations that occurred in‐person before the pandemic as well as evaluations that occurred via telehealth during the pandemic.

**TABLE 2:** Multinomial logistic regression model for association between timeframe (pandemic vs. pre‐pandemic) and diagnostic certainty.

	Not assessed (vs. no)			Possible (vs. no)			Yes (vs. no)		
	RRR	CI	*p*	RRR	CI	*p*	RRR	CI	*p*
ASD	—	—	—	3.67	(3.02, 4.32)	<0.001	0.90	(0.59, 1.2)	0.49
IDD	1.85	(1.54, 2.16)	<0.001	1.30	(0.91, 1.70)	0.18	0.55	(0.15, 0.95)	<0.001
ADHD	0.96	(0.61, 1.30)	0.81	1.62	(1.25, 2.00)	0.01	1.36	(0.93, 1.78)	0.16
ANX	1.02	(0.67, 1.37)	0.92	1.07	(0.71, 1.44)	0.70	1.00	(0.64, 1.37)	0.99
DEP	0.89	(0.62, 1.16)	0.41	3.26	(2.44, 4.08)	<0.001	2.28	(1.41, 3.14)	0.06
BD	0.77	(0.43, 1.10)	0.12	1.40	(0.92, 1.89)	0.17	1.41	(1.07, 1.74)	0.05

*Note*: Estimates are adjusted for age, sex, race, and insurance type. *p*‐values calculated using Wald 2‐tailed Z‐test.

Abbreviations: ADHD, attention‐deficit/hyperactivity disorder; ANX, anxiety; ASD, autism spectrum disorder; BD, behavioral disorder; DEP, depression; IDD, intellectual developmental disorder/global developmental delay; RRR, relative risk ratio.

### Evaluation methods

Final CBE diagnoses for all conditions explored were informed by developmental and medical history obtained from caregiver interview and review of medical and school records (e.g., individualized education programs, testing), caregiver report of behavior via interview and standardized rating scales, and direct child observation. Additionally, an electronic link leading to a teacher questionnaire about learning and behavior was routinely sent to each family to forward to the child's teacher; however, this information was inconsistently received back from the teacher across cohorts. Importantly, the interviews with families conducted via telehealth during the pandemic grossly reflected how these interviews were conducted in person pre‐pandemic, with both the child and caregiver present.

Given that the evaluations included in this study were clinical in nature, a diverse range of assessment tools and evaluation methods were utilized by our clinical team based on clinical indication and provider expertise. Here we provide information about general trends in assessment tool use and evaluation approaches in our clinic for in‐person evaluations completed before the pandemic and telehealth evaluations completed during the pandemic for context; however, quantitative information about the frequency of assessment tools used and specific information about clinical decision‐making related to assessment tool use and evaluation approaches taken to inform diagnostic decisions were not available and is a limitation of this study.

Prior to the pandemic, psychologists and SLPs generally administered the Autism Diagnostic Observation Schedule, Second Edition (ADOS‐2; Lord et al., 2012) in person to inform ASD diagnostic decision‐making. For telehealth evaluations conducted during the pandemic, psychologists and SLPs generally used the TAP or a non‐standardized structured telehealth observation utilizing social presses similar to the presses administered during an in‐person ADOS‐2 administration instead. The TAP takes 10–20 min to administer and is intended for children under 36 month of age. The TAP includes 12 social activities or social bids, including opportunities for free play and physical play routines (e.g., peekaboo, tickling), and activities (e.g., bubbles, snack) that may prompt a child to request. After the TAP, the clinician rates the child's behavior on seven items pertaining to the presence and severity of symptoms of ASD to inform a clinical diagnosis. The non‐standardized assessment was typically done if the child was out of the age range of the TAP. For a description of the non‐standardized structured telehealth observation employed at our center, see Ludwig et al. (2022).

Before the pandemic, diagnoses of IDD were informed by observation of the child as well as cognitive/developmental assessment and questionnaire/interview‐based assessment of adaptive functioning conducted directly with the child and family and/or per review of school and medical records. For telehealth evaluations conducted during the pandemic, clinicians continued to utilize these methods and were obligated to shift to remote forms of cognitive assessment when direct testing was needed. Multiple methods of remote cognitive assessment were utilized based on clinical indication as well as comfort and expertise of clinicians across our clinic. Specifically, some psychologists shifted to remote versions of cognitive assessments previously administered in person such as the Wechsler Intelligence Scale for Children, Fourth Edition (WISC‐V; Wechsler, 2014), Wechsler Abbreviated Scale of Intelligence, Second Edition (WASI‐II; Wechsler, 2011), and the Differential Abilities Scales, Second Edition (DAS‐II; Elliott, 2007; see Peterson et al., 2021 for additional information about how remote testing was completed by psychologists at our center) given emerging literature about telehealth administration equivalency to in person administrations (Hamner et al., 2021; Harder et al., 2020; McDermott et al., 2023; Ng et al., 2023; Wright, 2020). Similarly, some physicians informally adapted developmental assessments previously done in person including the Clinical Linguistic and Auditory Milestone Scale (Capute et al., 1986) and they continued to utilize the CDC's Developmental Milestones to gauge developmental level.

The Child Behavior Checklist, Parent Report Form (Achenbach & Ruffle, 2000) was administered to all children at intake as this was standard of care at our center before and during the pandemic. Additional parent social‐emotional and behavioral rating scales were also utilized as clinically indicated to inform diagnostic decisions (e.g., NICHQ Vanderbilt Assessment Scale, Wolraich, 2002). While rating scales were administered both electronically and via paper form before the pandemic, rating scales were primarily administered electronically during the pandemic for evaluations conducted via telehealth.

## MEASURES

### Demographics

Demographic information including age, biological sex, race, and insurance type was extracted from the EMR. Although Hispanic is an ethnic category, the EMR lists Hispanic as a racial category, which is a limitation of this dataset. Insurance type was classified as public (i.e., Medical Assistance) or private.

### Diagnostic Classification Table (DCT)

After an ASD evaluation, the diagnosing provider (i.e., physician or psychologist) was required to complete the DCT by selecting “*Yes,” “No,” “Possible,”* or “*Not Assessed*” for the following DSM‐5 disorders: ASD, IDD (included global developmental delay and IDD), ADHD, ANX (included any DSM‐5 anxiety disorder), DEP (included any DSM‐5 depressive disorder), and BD (included any DSM‐5 disruptive, impulse‐control, and conduct disorder) to reflect the final CBE diagnostic determination. These disorders were examined given their high prevalence in children referred for ASD evaluation. When providers selected “*Yes*,” it meant the diagnosis was made, and when providers selected “*No*” it meant the diagnosis was ruled‐out. When “*Possible”* was selected, the clinical diagnosis was not made and further evaluation for that diagnosis was recommended. As such, we interpreted instances when providers selected “*Possible*” as reduced diagnostic certainty. “*Not Assessed”* indicated that the clinician did not evaluate for that particular diagnosis within the context of the evaluation.

### Data analysis

The frequency of diagnostic determinations was compared between evaluations occurring in‐person (*n* = 649) and via telehealth (*n* = 1,543). Multinomial logistic regression models were used to examine the association between evaluation cohort and the diagnostic determinations for each disorder. For each outcome, “*No”* served as the reference group. Each disorder was analyzed separately; co‐occurring conditions were not considered. Models were adjusted for age, sex, race, and insurance type given differences across cohort. Relative risk ratios (RRR) and 95% confidence intervals were estimated for all models. *p*‐values for regression models were calculated using 2‐tailed Wald Z‐test.

## RESULTS

### Neurodevelopmental conditions

**FIGURE 2:**
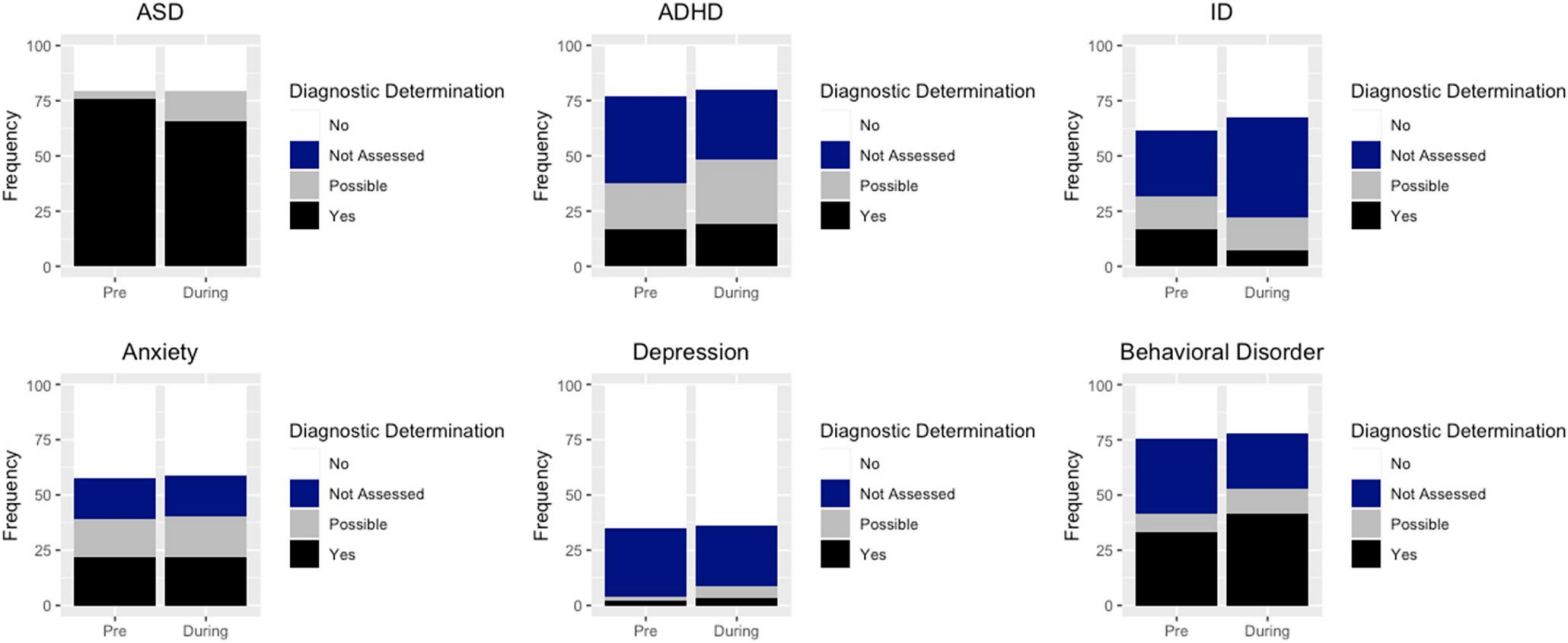
Frequency of assessment, diagnosis/rule‐out, and certainty of disorders based on the Diagnostic Classification Table completed at the end of the evaluation adjusted for covariates.

With regard to ASD, the likelihood of “*Possible”* (RRR = 3.67, 95% CI 3.02–4.32, *p* < 0.001) was more than three times higher for telehealth compared to in‐person evaluations, whereas the frequency of “*Yes”* (RRR = 0.90, 95% CI 0.59–1.20, *p* = 0.49) was similar across evaluation cohorts compared to “*No.*” With regard to ADHD, the likelihood of “*Possible”* (RRR = 1.62, 95% CI 1.25–2.00, *p* = 0.01) was higher for evaluations conducted via telehealth compared to in‐person evaluations, but the frequency of *“Not Assessed”* (RRR = 0.96, 95% CI 0.61–1.37, *p* = 0.92) and “*Yes”* (RRR = 1.36, 95% CI 0.93–1.78, *p* = 0.16) was similar across cohorts compared to “*No.*” With regard to IDD, the likelihood of “*Not Assessed”* (RRR = 1.85, 95% CI 1.54–2.16, *p* < 0.001) was higher and “*Yes”* (RRR = 0.55, 95% CI 0.15–0.96, *p* < 0.001) was lower for evaluations conducted via telehealth, whereas the frequency of “*Possible”* (RRR = 1.30, 95% CI 0.91–1.70, *p* = 0.18) was similar across cohorts compared to “*No*” (Table 2; Figure 2).

### Psychiatric disorders

For ANX, there were no differences in the frequency of “*Not Assessed*,” “*Possible*,” or “*Yes”* compared to “*No*” across evaluation cohorts (RRR's ranging from 1.00 to 1.07; all *p* > 0.05). With regard to DEP, the frequency of “*Possible”* (RRR = 3.26, 95% CI 2.44–4.08, *p* < 0.001) and “*Yes”* (RRR = 2.28, 95% CI 1.41–3.14, *p* = 0.06; trending) was higher, whereas there was not difference in the frequency of “*Not Assessed”* across evaluation cohort compared to “*No*” (RRR = 0.89, 95% CI 0.62–1.16, *p* = 0.41). Lastly, for BD, the frequency of “*Yes”* was higher (RRR = 1.41, 95% CI 1.07–1.74, *p* = 0.06), whereas there was no difference in the frequency of “*Not Assessed”* (RRR = 0.77 95% CI 0.43–1.10, *p* = 0.12) and “*Possible”* across cohort compared to “*No*” (RRR = 1.40 95% CI 0.92–1.89, *p* = 0.17; Table 2; Figure 2).

### Sensitivity analyses

While we did not generate a priori hypotheses about how results may differ based on patient‐level factors like sex and age, we conducted sensitivity analyses to explore potential trends. Results from these exploratory analyses (see Tables S1–S13) revealed some group‐level differences (e.g., males were diagnosed with ADHD more often, females were diagnosed with anxiety more often, and ASD diagnostic certainty was stable for the youngest children when the evaluation was conducted via telehealth during the pandemic compared to in person pre‐pandemic). Multinomial logistic regression models showing these associations are summarized in Tables S1–S6, while the unadjusted proportions of people across diagnostic groups are shown in Tables S1–S13. It should be noted that since Tables S7–S13 show raw, unadjusted percentages, as opposed to the adjusted regression estimates in Tables S1–S6, some inferences may be slightly different between the two sets of tables.

## DISCUSSION

This study is the first to examine differences in diagnostic patterns and diagnostic certainty among a large sample of children evaluated for ASD via telehealth during the pandemic compared to in person pre‐pandemic at an ASD specialty center. With regard to neurodevelopmental conditions, results show lower diagnostic certainty in making a diagnosis of ASD or ADHD and fewer evaluations of and diagnosis of IDD for evaluations conducted via telehealth during the pandemic compared to in person pre‐pandemic. For psychiatric conditions, the frequency of DEP and BD diagnoses was higher for evaluations conducted via telehealth during the pandemic compared to in person pre‐pandemic, while the frequency of ANX diagnoses remained the same across evaluation cohorts. These findings suggest neurodevelopmental diagnostic disparities may have arisen during the pandemic, as more children were referred for further evaluation of their ASD and ADHD symptoms after initial telehealth evaluation (i.e., diagnosis deferred) and more children who likely presented with IDD were not being evaluated and diagnosed via telehealth. Secondly, results suggest the pandemic may have contributed to more diagnoses of DEP and BP.

### Neurodevelopmental conditions

As hypothesized, diagnostic certainty was lower for ASD and ADHD. The limited experience clinicians had with diagnostic observation via telehealth (Kryszak et al., 2022) paired with a dearth of well‐established tools to facilitate observation at this point in time during the pandemic (i.e., though July 2021) likely contributed to this difference. Beyond limitations of the telehealth modality, it is also important to acknowledge the context of the pandemic itself may have also complicated the diagnostic process when considering diagnoses of ASD and ADHD. Specifically, most children likely had fewer opportunities to engage with peers due to social distancing restrictions during the pandemic, which may have limited the ability of the parent to comment on social difficulties with peers (pertaining to an ASD diagnosis) and/or attention problems in social contexts (pertaining to ADHD diagnosis).

There are additional reasons why diagnostic certainty of ADHD may have been lower for evaluations conducted via telehealth during the pandemic. Specifically, the opportunity to obtain report of symptoms in multiple settings, a requirement for the diagnosis, was limited by the transition to virtual schooling at home. The shift to virtual instruction changed the way in which teachers observed symptoms given the lack of in‐person interactions. This may have limited the ability to confirm the presence of ADHD symptoms across settings and contributed to lower diagnostic certainty. Beyond observation, increased situational stressors impacting attention associated with the pandemic (e.g., extended hours on a computer screen during virtual schooling, onset/exacerbation of other mental health conditions, reduced sleep (Panchal et al., 2021), and confinement in general (Giménez‐Dasí et al., 2020), versus pervasive attention symptoms reflective of ADHD was likely an added challenge for clinicians during the pandemic (Stein, 2022)).

Taken together, there were likely both telehealth factors and pandemic factors contributing to lower diagnostic certainty in making ASD and ADHD diagnoses during the pandemic. Despite a similar frequency of ADHD evaluations across evaluation cohorts, it is important to emphasize that lower diagnostic certainty meant that more children were identified as possibly having ASD and/or ADHD without a diagnosis being made or ruled out (i.e., diagnosis was deferred). This yielded more children requiring follow up later, thereby delaying confirmatory diagnostic status and contributing to a backlog of patients needing in‐person services later. This ultimately could have delayed the delivery of intervention for those requiring re‐evaluation and may have delayed time to evaluation and diagnosis for new referrals. Given the importance of early identification and treatment for both ASD and ADHD (Landa, 2018; Shaw et al., 2012), the ramifications of more frequent deferred diagnoses during the pandemic will likely have implications on developmental and behavioral outcomes at our center.

While diagnostic certainty of IDD was equivalent across cohorts, results suggest that IDD was evaluated less often and fewer diagnoses were made via telehealth. Telehealth assessment of intellectual ability was not common practice prior to the pandemic. In fact, a survey of neuropsychologists attending a webinar about remote cognitive testing early in the pandemic (i.e., April 2020) indicated that only 11% of providers had used telehealth for remote testing prior to the pandemic (Hammers et al., 2020). There are many practical and ethical factors to consider in adapting cognitive testing for telehealth including technical, conceptual, and psychometric issues (Van Patten, 2021). Indeed, Hammers and colleagues' survey also indicated that only 44% of neuropsychologists felt comfortable with the ethics of remote testing. Given the challenges of transitioning cognitive testing to a telehealth modality, the Neuropsychology InterOrganizational Practice Committee (IOPC) provided a position statement in July 2020 in an effort to provide guidelines related to remote testing; however, the authors stated that there was no clear guidance about how to modify current tests for remote administration and threats to validity that must be considered (Bilder et al., 2020). As a result, there are significant barriers to transitioning cognitive testing to telehealth that likely contributed to lower frequency of evaluation for IDD and lower frequency of diagnoses made via telehealth compared to in‐person evaluation at our ASD center. It is important to recognize that the validity of telehealth intellectual assessment has not yet been established by test developers and this is a limitation. However, it is unlikely that the reduced frequency of IDD diagnoses via telehealth was due to an under‐detection of low IQ when testing was administered via telehealth given emerging evidence of telehealth equivalency (Hamner et al., 2021; Harder et al., 2020; McDermott et al., 2023; Ng et al., 2023; Wright, 2020). Instead, this fact may have impacted examiner confidence in using these tools during evaluation, thereby impacting frequency of assessment for and diagnosis of IDD. Beyond cognitive testing as a barrier, providers often utilize information gathered from school (i.e., teachers, therapists) to support a diagnosis of IDD (Patel et al., 2020). This also may have impacted evaluation and diagnosis of IDD as schooling was primarily virtual in our region during the interval of focus in the present study, thus the nature of these observations was inherently different from when children physically attended school. Nonetheless, like ASD and ADHD, fewer evaluations of and diagnosis of IDD via telehealth during the pandemic means that there are likely children with IDD that were not diagnosed during the pandemic, which may have delayed access to intervention and services shown helpful in improving developmental outcomes in this group (Guralnick, 2017).

### Psychiatric conditions

There was no difference in diagnostic certainty of ANX across evaluation cohorts, which is consistent with the idea that diagnosis of psychiatric conditions are relatively reliant on report of symptoms rather than observation, and therefore may not be as impacted by the shift to telehealth assessment. Surprisingly though, the frequency of evaluation and diagnosis of ANX was not different across evaluation cohorts as well, contrary to evidence of increased ANX symptoms in children more broadly during the pandemic (Walsh et al., 2021). One possible explanation is that exacerbation of ANX symptoms did not result in differences in prevalence of ANX disorders during the pandemic. These symptoms may have remained sub‐threshold to a clinical diagnosis or may have been reflected in other diagnoses. For example, a diagnosis of adjustment disorder (listed as a trauma‐ and stressor‐related disorder rather than an ANX disorder in the DSM‐5), requires emotional/behavioral distress in response to an identifiable stressor, which may have been utilized by practitioners to capture ANX associated with the pandemic instead of an ANX disorder. This is consistent with increased prevalence of adjustment disorder during the pandemic (Dragan et al., 2021). Another possibility is the potential reduction of social stressors with being at home for children with ASD, leading to improvement in ANX symptoms for some and resulting in no net change in diagnosis (Reicher, 2020). Indeed, Vasa and colleagues demonstrated similar or improved ANX symptoms in children with ASD during the pandemic for the majority of children with a pre‐existing ANX diagnoses (Vasa et al., 2021).

As hypothesized, we found that diagnoses of DEP and BD were provided more often during the pandemic. This is consistent with a recent systematic review citing increased symptoms of DEP as one of the most common outcomes during the pandemic (Panchal et al., 2021) as well as literature demonstrating children with neurodevelopmental disabilities (NDDs) showed more conduct problems during the pandemic (Nonweiler et al., 2020). Notably, some research has shown that increased behavioral problems for children with NDDs is related to decreased access to therapy during the pandemic (Bentenuto et al., 2021), highlighting the crucial nature of these interventions for this group.

Lower diagnostic certainty of DEP via telehealth observed in this study may be due to difficulties with symptom assessment within the context of the pandemic. Specifically, a diagnosis of DEP typically involves evaluating the degree to which someone is interested in activities they enjoy (i.e., motivation); however, during the pandemic, many people could not safely engage in activities they enjoyed, potentially making it difficult to tease apart decreased interest and decreased participation due to pandemic‐related issues. Weight changes are also indicative of DEP, which may be confounded by increased weight due to pandemic‐related lifestyle changes (Chang et al., 2021). Concentration difficulty is also a symptom of DEP, which was also exacerbated broadly for many children during the pandemic as discussed above. Taken together, the task of assessing DEP symptoms during the pandemic when children were not in their regular routines, may have lowered diagnostic certainty.

### Strengths and limitations

The present study has many strengths and several notable limitations. For strengths, the sample was large, with continuous data collection before and during the pandemic, community‐based, and both socio‐demographically and clinically heterogeneous. Most importantly, our findings reflect real‐world practice as we gathered information about diagnostic patterns on a range of disorders directly from specialty clinicians.

For limitations, due to the abrupt shift to telehealth, our clinic was unable to quantify several important variables that could have been useful in further interpreting results across cohorts. For example, whether the evaluation was conducted by a single provider or team, composition of the team, length of visits, use of specific assessment tools, scores on assessment tools, information about the specific data sources used to make a diagnosis (e.g., was cognitive assessment data collected during the evaluation or was school testing used), and whether the evaluation was conducted in a language other than English/if an interpreter was used. Differences in these factors across cohorts could contribute to variability in diagnostic patterns and diagnostic certainty and warrant future inquiry.

Furthermore, there are some questions that are beyond the scope of this study, but warrant future exploration. For example, we were unable to explore the unique effect of having a co‐occurring condition on diagnostic certainty (e.g., ASD within the context of IDD or ADHD or both) given the majority of the sample demonstrated at least one co‐occurring condition (i.e., 65%–90% depending on the condition of interest). When regression models were run with only participants with co‐occurring condition, findings were consistent with those run with the whole sample (Table S14). Future work should explore the impact of co‐occurring conditions in a sample with a higher proportion of individuals without a co‐occurring condition. Additionally, patient‐level factors including sex and age are also important to consider (Tables S1–S13). While we adjusted for these particular variables in our primary models, future work to understand the nuances of how these and other patient factors, such as child language level and cognitive level, influence and interact with diagnostic certainty will be important for informing diagnostic approaches.

There are several design limitations that also must be considered. Diagnostic certainly was not explicitly measured in this study, but was inferred based on endorsement of “possible” on the DCT. While we believe this is a good proxy for certainly, this should be considered when interpreting findings. Additionally, although it would have been ideal to have equal sample sizes and a similar time‐frame across evaluation cohorts, our in‐person sample was smaller and representative of a shorter time‐frame than the telehealth cohort given that the DCT was only implemented within a year prior to the start of the pandemic, and as such, more data were not available. Further, this study was limited to assessments conducted through July 2021; however, new diagnostic tools to facilitate telehealth assessment for neurodevelopmental disorders have been introduced and are being refined over time. Those tools may impact assessment and diagnostic approaches via telehealth moving forward, which should be considered, although community adoption of evidenced‐based methods is often slow. We were also unable to elucidate the potential unique impact of pandemic factors and telehealth factors on diagnostic patterns with the current study design and because the study compares two cohorts at different points in time, the ability to compare these samples directly is limited. While there may be inherent differences in the cohorts, we attempted to address this issue by controlling for differences in demographics that emerged across time‐point and assessment modality (i.e., age, sex, race, insurance type). Nevertheless, there may still be other differences across samples that are specific to the impact of the pandemic. For example, stress associated with the pandemic, reduced opportunities for socialization, quality of therapies and educational opportunities are just some of the differences that may have differentially impacted our cohorts and the outcomes. While these factors likely did not have a strong impact the true prevalence of neurodevelopmental disorders (i.e., ASD, ADHD, or IDD) during the pandemic, given the relatively strong genetic basis for these conditions, they could have impacted the detection of these disorders within the context of an evaluation (i.e., made these diagnoses less clear/salient, difficult to diagnose). Taken together, we feel that the clinical differences observed across cohorts were not only attributed to evaluation approach (i.e., in‐person or telehealth), but also impacted by the patients themselves due to pandemic‐related factors and it is difficult to tease apart the unique contribution of these factors with the current study design.

Findings must also be considered within the context of the way in which the sample was ascertained. Participants were children referred for an ASD evaluation based on concerns about ASD, and many of the individuals referred are ultimately diagnosed with ASD (Table 2). As such, these findings may not generalize to other clinics where ASD evaluation was not the primary focus of evaluation. Finally, this study was completed at one ASD specialty center and multi‐center studies are warranted in order to understand generalizability of findings.

Despite these important limitations, our findings reflect clinical changes that occurred at our center within the context of telehealth evaluations during the pandemic that are of clinical importance. As such, we hope our initial findings presented here foster further inquiry to understand the generalizability of these patterns and factors that may have contributed to these clinically meaningful differences.

## CONCLUSIONS

In sum, we observed differences in diagnoses evaluated and made as well as diagnostic certainty during evaluations conducted via telehealth during the pandemic and those conducted in‐person before the pandemic in children evaluated for ASD at our specialty center. We observed lower certainty of ASD and ADHD diagnoses and fewer evaluations of and diagnosis of IDD via telehealth, meaning these diagnoses may have been delayed for some children. In contrast, we observed more diagnosis of some psychiatric conditions via telehealth, which is consistent with other research that has demonstrated more mental health distress during the pandemic. Our data suggest that telehealth may be capturing these concerns, at least for DEP and BD, which is promising.

## AUTHOR CONTRIBUTIONS

**Natasha N. Ludwig**: Conceptualization; methodology; project administration; writing—original draft; writing—review and editing. **Calliope Holingue**: Conceptualization; data curation; formal analysis; methodology; visualization; writing—original draft; writing—review and editing. **Ji Su Hong**: Conceptualization; investigation; methodology; writing—review and editing. **Luther Kalb**: Conceptualization; methodology; writing—review and editing. **Danika Pfeiffer**: Conceptualization; methodology; writing—review and editing. **Rachel Reetzke**: Conceptualization; investigation; methodology; writing—review and editing. **Deepa Menon**: Conceptualization; investigation; methodology; writing—review and editing. **Rebecca Landa**: Funding acquisition; methodology; resources; supervision; writing—review and editing.

## CONFLICT OF INTEREST STATEMENT

The authors declare no conflicts of interest.

## Ethical considerations

A total of 649 children who received an in‐person diagnostic evaluation between September 1, 2019 and March 13, 2020 (pre‐pandemic) and 1543 who received a telehealth diagnostic evaluation between March 14, 2020 and July 26, 2021 (during pandemic) were included in this study. Children's data were included if their parent/guardian consented to participate in the center's institutional review board‐approved clinical research registry (IRB NA_00010880). The consent rate for the registry is approximately 80% (Kalb et al., 2019).

## Supporting information

Supporting Information S1

## Data Availability

The data is not publicly available due to privacy or ethical restrictions. The data that support the findings of this study are available on reasonable request from the corresponding author.
