# Supplementary material for: Diagnostic certainty during in‐person and telehealth autism evaluations
Source: JCPP Adv. 2023 Oct 28;4(1):e12201. doi: 10.1002/jcv2.12201 (PMC10933595; doi:10.1002/jcv2.12201)
Supplement: Supplementary file 1 — Supporting Information S1 [file JCV2-4-e12201-s001.docx]

Supporting Information

Table S1

*Multinomial Logistic Regression Model for Association between Timeframe (Pandemic versus Pre-Pandemic) and Diagnostic Certainty, Among Males*

|  | Not Assessed (versus No) | | | Possible (versus No) | | | Yes (versus No) | | |
| --- | --- | --- | --- | --- | --- | --- | --- | --- | --- |
|  | RRR | CI | P | RRR | CI | P | RRR | CI | P |
| ASD | --- | --- | --- | 4.34 | (3.59 , 5.09) | <.001 | 0.95 | (0.60 , 1.29) | 0.76 |
| IDD | 2.07 | (1.73 , 2.42) | <.001 | 1.29 | (0.86 , 1.72) | 0.24 | 0.62 | (0.17 , 1.07) | 0.04 |
| ADHD | 1.10 | (0.71 , 1.48) | 0.63 | 1.94 | (1.51 , 2.36) | <.001 | 1.69 | (1.21 , 2.17) | 0.03 |
| Anxiety | 0.88 | (0.50 , 1.27) | 0.53 | 0.99 | (0.59 , 1.39) | 0.96 | 0.85 | (0.44 , 1.26) | 0.44 |
| Depression | 0.84 | (0.54 , 1.14) | 0.24 | 2.67 | (1.78 , 3.55) | 0.03 | 2.18 | (1.13 , 3.24) | 0.15 |
| Behavioral Disorder | 0.80 | (0.42 , 1.18) | 0.26 | 1.42 | (0.88 , 1.97) | 0.21 | 1.33 | (0.96 , 1.71) | 0.13 |

*Note.* Estimates are adjusted for age, race, and insurance type. P-values calculated using Wald 2-tailed z-test. RRR=relative risk ratio.

Table S2

*Multinomial Logistic Regression Model for Association between Timeframe (Pandemic versus Pre-Pandemic) and Diagnostic Certainty, Among Females*

|  | Not Assessed (versus No) | | | Possible (versus No) | | | Yes (versus No) | | |
| --- | --- | --- | --- | --- | --- | --- | --- | --- | --- |
|  | RRR | CI | P | RRR | CI | P | RRR | CI | P |
| ASD | --- | --- | --- | 2.17 | (0.81 , 3.52) | 0.26 | 0.73 | (0.03 , 1.43) | 0.38 |
| IDD | 1.21 | (0.50 , 1.92) | 0.61 | 1.39 | (0.38 , 2.40) | 0.53 | 0.33 | (-0.61 , 1.28) | 0.02 |
| ADHD | 0.52 | (-0.31 , 1.36) | 0.13 | 0.80 | (-0.10 , 1.70) | 0.63 | 0.51 | (-0.49 , 1.51) | 0.18 |
| Anxiety | 2.02 | (1.11 , 2.93) | 0.13 | 1.64 | (0.79 , 2.49) | 0.26 | 2.11 | (1.26 , 2.95) | 0.08 |
| Depression | 1.25 | (0.60 , 1.91) | 0.50 | 7.69 | (5.48 , 9.90) | 0.07 | 2.24 | (0.66 , 3.82) | 0.32 |
| Behavioral Disorder | 0.58 | (-0.16 , 1.32) | 0.15 | 1.37 | (0.31 , 2.43) | 0.56 | 1.77 | (0.97 , 2.57) | 0.16 |

*Note.* Estimates are adjusted for age, race, and insurance type. P-values calculated using Wald 2-tailed z-test. RRR=relative risk ratio.

Table S3

*Multinomial Logistic Regression Model for Association between Timeframe (Pandemic versus Pre-Pandemic) and Diagnostic Certainty, Among Individuals Less than 3 Years Old*

|  | Not Assessed (versus No) | | | Possible (versus No) | | | Yes (versus No) | | |
| --- | --- | --- | --- | --- | --- | --- | --- | --- | --- |
|  | RRR | CI | P | RRR | CI | P | RRR | CI | P |
| ASD | --- | --- | --- | 2.98 | (1.53 , 4.43) | 0.14 | 0.98 | (0.16 , 1.81) | 0.97 |
| IDD | 1.24 | (0.48 , 2.00) | 0.58 | 0.54 | (-0.45 , 1.52) | 0.21 | 0.22 | (-0.96 , 1.40) | 0.01 |
| ADHD | 2.45 | (1.68 , 3.22) | 0.02 | 1.21 | (0.42 , 1.99) | 0.64 | 0.18 | (-1.28 , 1.64) | 0.02 |
| Anxiety | 0.36 | (-0.63 , 1.35) | 0.04 | 0.28 | (-0.58 , 1.14) | 0.00 | 0.26 | (-0.75 , 1.26) | 0.01 |
| Depression | --- | --- | --- | --- | --- | --- | --- | --- | --- |
| Behavioral Disorder | 2.14 | (0.92 , 3.35) | 0.22 | 1.19 | (-0.02 , 2.39) | 0.78 | 1.48 | (0.80 , 2.16) | 0.25 |

*Note.* Estimates are adjusted for age, sex, race, and insurance type. P-values calculated using Wald 2-tailed z-test. RRR=relative risk ratio.

Table S4

*Multinomial Logistic Regression Model for Association between Timeframe (Pandemic versus Pre-Pandemic) and Diagnostic Certainty, Among Individuals 3-5 Years Old*

|  | Not Assessed (versus No) | | | Possible (versus No) | | | Yes (versus No) | | |
| --- | --- | --- | --- | --- | --- | --- | --- | --- | --- |
|  | RRR | CI | P | RRR | CI | P | RRR | CI | P |
| ASD |  | --- | --- | 2.42 | (1.43 , 3.42) | 0.08 | 1.00 | (0.44 , 1.56) | 0.99 |
| IDD | 1.46 | (0.89 , 2.03) | 0.19 | 0.92 | (0.30 , 1.54) | 0.79 | 0.74 | (-0.02 , 1.49) | 0.43 |
| ADHD | 1.08 | (0.50 , 1.66) | 0.79 | 1.50 | (0.87 , 2.12) | 0.21 | 1.01 | (0.18 , 1.84) | 0.98 |
| Anxiety | 0.79 | (0.27 , 1.31) | 0.38 | 1.02 | (0.39 , 1.66) | 0.94 | 1.05 | (0.26 , 1.83) | 0.90 |
| Depression | 0.51 | (0.06 , 0.96) | <.001 | --- | --- | --- | --- | --- | 0.00 |
| Behavioral Disorder | 1.25 | (0.59 , 1.91) | 0.50 | 1.21 | (0.39 , 2.03) | 0.65 | 2.20 | (1.60 , 2.8) | 0.01 |

*Note.* Estimates are adjusted for age, sex, race, and insurance type. P-values calculated using Wald 2-tailed z-test. RRR=relative risk ratio.

Table S5

*Multinomial Logistic Regression Model for Association between Timeframe (Pandemic versus Pre-Pandemic) and Diagnostic Certainty, Among Individuals 6-11 Years Old*

|  | Not Assessed (versus No) | | | Possible (versus No) | | | Yes (versus No) | | |
| --- | --- | --- | --- | --- | --- | --- | --- | --- | --- |
|  | RRR | CI | P | RRR | CI | P | RRR | CI | P |
| ASD | --- | --- | --- | 4.59 | (3.32 , 5.86) | 0.02 | 0.74 | (0.22 , 1.27) | 0.27 |
| IDD | 1.98 | (1.46 , 2.49) | 0.01 | 3.47 | (2.59 , 4.36) | 0.01 | 0.90 | (0.19 , 1.60) | 0.76 |
| ADHD | 0.82 | (0.08 , 1.56) | 0.61 | 2.97 | (2.17 , 3.78) | 0.01 | 2.33 | (1.56 , 3.10) | 0.03 |
| Anxiety | 3.07 | (2.37 , 3.77) | <.001 | 1.75 | (1.15 , 2.35) | 0.07 | 1.44 | (0.88 , 2.01) | 0.20 |
| Depression | 2.23 | (1.73 , 2.73) | <.001 | 2.99 | (1.88 , 4.10) | 0.05 | 7.10 | (5.04 , 9.16) | 0.06 |
| Behavioral Disorder | 0.66 | (0.09 , 1.24) | 0.16 | 1.64 | (0.79 , 2.48) | 0.25 | 1.36 | (0.71 , 2.01) | 0.36 |

*Note.* Estimates are adjusted for age, sex, race, and insurance type. P-values calculated using Wald 2-tailed z-test. RRR=relative risk ratio.

Table S6

*Multinomial Logistic Regression Model for Association between Timeframe (Pandemic versus Pre-Pandemic) and Diagnostic Certainty, Among Individuals 12 or More Years Old*

|  | Not Assessed (versus No) | | | Possible (versus No) | | | Yes (versus No) | | |
| --- | --- | --- | --- | --- | --- | --- | --- | --- | --- |
|  | RRR | CI | P | RRR | CI | P | RRR | CI | P |
| ASD | --- | --- | --- | --- | --- | <.001 | 1.08 | (0.30 , 1.86) | 0.84 |
| IDD | 5.19 | (3.89 , 6.49) | 0.01 | 5.32 | (3.20 , 7.44) | 0.12 | 0.29 | (-0.69 , 1.28) | 0.01 |
| ADHD | 0.31 | (-1.31 , 1.92) | 0.15 | 0.56 | (-1.22 , 2.33) | 0.52 | 0.44 | (-1.22 , 2.09) | 0.33 |
| Anxiety | 2.12 | (0.71 , 3.52) | 0.29 | 6.57 | (5.22 , 7.93) | 0.01 | 3.73 | (2.74 , 4.72) | 0.01 |
| Depression | 2.84 | (1.73 , 3.95) | 0.07 | 4.30 | (3.11 , 5.50) | 0.02 | 1.73 | (0.63 , 2.82) | 0.33 |
| Behavioral Disorder | 0.62 | (-0.25 , 1.48) | 0.27 | 5.28 | (3.08 , 7.47) | 0.14 | 0.89 | (-0.25 , 2.02) | 0.84 |

*Note.* Estimates are adjusted for age, sex, race, and insurance type. P-values calculated using Wald 2-tailed z-test. RRR=relative risk ratio.

Table S7

*Proportions of People Across Diagnostic Certainty Categories (Pandemic versus Pre-Pandemic), Among Males*

|  |  | Pre-Pandemic | During pandemic |
| --- | --- | --- | --- |
| ASD | No | 33% | 67% |
|  | Possible | 16% | 84% |
|  | Yes | 33% | 67% |
|  |  | Pre-Pandemic | During pandemic |
| IDD | No | 38% | 62% |
|  | Not Assessed | 22% | 78% |
|  | Possible | 31% | 69% |
|  | Yes | 45% | 55% |
|  |  | Pre-Pandemic | During pandemic |
| ADHD | No | 30% | 70% |
|  | Not Assessed | 34% | 66% |
|  | Possible | 30% | 70% |
|  | Yes | 28% | 72% |
|  |  | Pre-Pandemic | During pandemic |
| Anxiety | No | 29% | 71% |
|  | Not Assessed | 35% | 65% |
|  | Possible | 31% | 69% |
|  | Yes | 32% | 68% |
|  |  | Pre-Pandemic | During pandemic |
| Depression | No | 31% | 69% |
|  | Not Assessed | 34% | 66% |
|  | Possible | 23% | 77% |
|  | Yes | 31% | 69% |
|  |  | Pre-Pandemic | During pandemic |
| Behavioral Disorder | No | 34% | 66% |
|  | Not Assessed | 39% | 61% |
|  | Possible | 24% | 76% |
|  | Yes | 26% | 74% |

Table S8

*Proportions of People Across Diagnostic Certainty Categories (Pandemic versus Pre-Pandemic), Among Females*

|  |  | Pre-Pandemic | During pandemic |
| --- | --- | --- | --- |
| ASD | No | 23% | 77% |
|  | Possible | 10% | 90% |
|  | Yes | 26% | 74% |
|  |  | Pre-Pandemic | During pandemic |
| IDD | No | 26% | 74% |
|  | Not Assessed | 21% | 79% |
|  | Possible | 14% | 86% |
|  | Yes | 43% | 57% |
|  |  | Pre-Pandemic | During pandemic |
| ADHD | No | 19% | 81% |
|  | Not Assessed | 29% | 71% |
|  | Possible | 20% | 80% |
|  | Yes | 28% | 73% |
|  |  | Pre-Pandemic | During pandemic |
| Anxiety | No | 23% | 77% |
|  | Not Assessed | 22% | 78% |
|  | Possible | 24% | 76% |
|  | Yes | 25% | 75% |
|  |  | Pre-Pandemic | During pandemic |
| Depression | No | 23% | 77% |
|  | Not Assessed | 28% | 72% |
|  | Possible | 17% | 83% |
|  | Yes | 19% | 81% |
|  |  | Pre-Pandemic | During pandemic |
| Behavioral Disorder | No | 24% | 76% |
|  | Not Assessed | 36% | 64% |
|  | Possible | 24% | 76% |
|  | Yes | 15% | 85% |

Table S9

*Proportions of People Across Diagnostic Certainty Categories (Pandemic versus Pre-Pandemic), Among Individuals Less than 3 Years Old*

|  |  | Pre-Pandemic | During pandemic |
| --- | --- | --- | --- |
| ASD | No | 25% | 75% |
|  | Possible | 20% | 80% |
|  | Yes | 23% | 77% |
|  |  | Pre-Pandemic | During pandemic |
| IDD | No | 23% | 77% |
|  | Not Assessed | 17% | 83% |
|  | Possible | 34% | 66% |
|  | Yes | 52% | 48% |
|  |  | Pre-Pandemic | During pandemic |
| ADHD | No | 24% | 76% |
|  | Not Assessed | 17% | 83% |
|  | Possible | 27% | 73% |
|  | Yes | 50% | 50% |
|  |  | Pre-Pandemic | During pandemic |
| Anxiety | No | 19% | 81% |
|  | Not Assessed | 36% | 64% |
|  | Possible | 33% | 67% |
|  | Yes | 37% | 63% |
|  |  | Pre-Pandemic | During pandemic |
| Depression | No | 20% | 80% |
|  | Not Assessed | 38% | 62% |
|  | Possible | --- | --- |
|  | Yes | --- | --- |
|  |  | Pre-Pandemic | During pandemic |
| Behavioral Disorder | No | 23% | 77% |
|  | Not Assessed | 22% | 78% |
|  | Possible | 21% | 79% |
|  | Yes | 23% | 77% |

Table S10

*Proportions of People Across Diagnostic Certainty Categories (Pandemic versus Pre-Pandemic), Among Individuals 3-5 Years Old*

|  |  | Pre-Pandemic | During pandemic |
| --- | --- | --- | --- |
| ASD | No | 27% | 73% |
|  | Possible | 16% | 84% |
|  | Yes | 30% | 70% |
|  |  | Pre-Pandemic | During pandemic |
| IDD | No | 36% | 64% |
|  | Not Assessed | 21% | 79% |
|  | Possible | 29% | 71% |
|  | Yes | 39% | 61% |
|  |  | Pre-Pandemic | During pandemic |
| ADHD | No | 27% | 73% |
|  | Not Assessed | 26% | 74% |
|  | Possible | 29% | 71% |
|  | Yes | 34% | 66% |
|  |  | Pre-Pandemic | During pandemic |
| Anxiety | No | 26% | 74% |
|  | Not Assessed | 37% | 63% |
|  | Possible | 26% | 74% |
|  | Yes | 20% | 80% |
|  |  | Pre-Pandemic | During pandemic |
| Depression | No | 23% | 77% |
|  | Not Assessed | 37% | 63% |
|  | Possible | 33% | 67% |
|  | Yes | 0% | 100% |
|  |  | Pre-Pandemic | During pandemic |
| Behavioral Disorder | No | 36% | 64% |
|  | Not Assessed | 34% | 66% |
|  | Possible | 31% | 69% |
|  | Yes | 23% | 77% |

Table S11

*Proportions of People Across Diagnostic Certainty Categories (Pandemic versus Pre-Pandemic), Among Individuals 6-11 Years Old*

|  |  | Pre-Pandemic | During pandemic |
| --- | --- | --- | --- |
| ASD | No | 32% | 68% |
|  | Possible | 11% | 89% |
|  | Yes | 37% | 63% |
|  |  | Pre-Pandemic | During pandemic |
| IDD | No | 38% | 63% |
|  | Not Assessed | 26% | 74% |
|  | Possible | 24% | 76% |
|  | Yes | 46% | 54% |
|  |  | Pre-Pandemic | During pandemic |
| ADHD | No | 38% | 62% |
|  | Not Assessed | 46% | 54% |
|  | Possible | 29% | 71% |
|  | Yes | 25% | 75% |
|  |  | Pre-Pandemic | During pandemic |
| Anxiety | No | 41% | 59% |
|  | Not Assessed | 26% | 74% |
|  | Possible | 34% | 66% |
|  | Yes | 32% | 68% |
|  |  | Pre-Pandemic | During pandemic |
| Depression | No | 39% | 61% |
|  | Not Assessed | 28% | 72% |
|  | Possible | 21% | 79% |
|  | Yes | 18% | 82% |
|  |  | Pre-Pandemic | During pandemic |
| Behavioral Disorder | No | 36% | 64% |
|  | Not Assessed | 42% | 58% |
|  | Possible | 23% | 77% |
|  | Yes | 25% | 75% |

Table S12

*Proportions of People Across Diagnostic Certainty Categories (Pandemic versus Pre-Pandemic), Among Individuals 12 or More Years Old*

|  |  | Pre-Pandemic | During pandemic |
| --- | --- | --- | --- |
| ASD | No | 38% | 63% |
|  | Possible | 4% | 96% |
|  | Yes | 37% | 63% |
|  |  | Pre-Pandemic | During pandemic |
| IDD | No | 39% | 61% |
|  | Not Assessed | 20% | 80% |
|  | Possible | 11% | 89% |
|  | Yes | 49% | 51% |
|  |  | Pre-Pandemic | During pandemic |
| ADHD | No | 32% | 68% |
|  | Not Assessed | 45% | 55% |
|  | Possible | 24% | 76% |
|  | Yes | 27% | 73% |
|  |  | Pre-Pandemic | During pandemic |
| Anxiety | No | 53% | 47% |
|  | Not Assessed | 28% | 72% |
|  | Possible | 20% | 80% |
|  | Yes | 33% | 67% |
|  |  | Pre-Pandemic | During pandemic |
| Depression | No | 45% | 55% |
|  | Not Assessed | 18% | 83% |
|  | Possible | 22% | 78% |
|  | Yes | 31% | 69% |
|  |  | Pre-Pandemic | During pandemic |
| Behavioral Disorder | No | 29% | 71% |
|  | Not Assessed | 46% | 54% |
|  | Possible | 12% | 88% |
|  | Yes | 24% | 76% |

Table S13

*Proportions of People Across Diagnostic Certainty Categories (Pandemic versus Pre-Pandemic), Among Individuals with One or More Comorbidities*

|  |  | Pre-Pandemic | During pandemic |
| --- | --- | --- | --- |
| ASD | No | 31% | 69% |
|  | Possible | 12% | 88% |
|  | Yes | 31% | 69% |
|  |  | Pre-Pandemic | During pandemic |
| IDD | No | 36% | 64% |
|  | Not Assessed | 21% | 79% |
|  | Possible | 29% | 71% |
|  | Yes | 45% | 55% |
|  |  | Pre-Pandemic | During pandemic |
| ADHD | No | 27% | 73% |
|  | Not Assessed | 34% | 66% |
|  | Possible | 29% | 71% |
|  | Yes | 27% | 73% |
|  |  | Pre-Pandemic | During pandemic |
| Anxiety | No | 28% | 72% |
|  | Not Assessed | 33% | 67% |
|  | Possible | 31% | 69% |
|  | Yes | 29% | 71% |
|  |  | Pre-Pandemic | During pandemic |
| Depression | No | 30% | 70% |
|  | Not Assessed | 33% | 67% |
|  | Possible | 21% | 79% |
|  | Yes | 27% | 73% |
|  |  | Pre-Pandemic | During pandemic |
| Behavioral Disorder | No | 33% | 67% |
|  | Not Assessed | 40% | 60% |
|  | Possible | 25% | 75% |
|  | Yes | 25% | 75% |

Table S14

*Multinomial Logistic Regression Model for Association between Timeframe (Pandemic versus Pre-Pandemic) and Diagnostic Certainty, Among Individuals with One or More Comorbidities*

|  | Not Assessed (versus No) | | | Possible (versus No) | | | Yes (versus No) | | |
| --- | --- | --- | --- | --- | --- | --- | --- | --- | --- |
|  | RRR | CI | P | RRR | CI | P | RRR | CI | P |
| ASD | --- | --- | --- | 4.65 | (3.82 , 5.49) | <.001 | 0.96 | (0.59 , 1.33) | 0.84 |
| IDD | 1.82 | (1.49 , 2.15) | <.001 | 1.10 | (0.69 , 1.52) | 0.64 | 0.54 | (0.13 , 0.95) | <.001 |
| ADHD | 0.96 | (0.60 , 1.32) | 0.83 | 1.64 | (1.25 , 2.04) | 0.01 | 1.50 | (1.05 , 1.96) | 0.08 |
| Anxiety | 0.95 | (0.58 , 1.31) | 0.77 | 1.00 | (0.62 , 1.38) | 0.99 | 1.09 | (0.69 , 1.48) | 0.67 |
| Depression | 0.86 | (0.57 , 1.14) | 0.29 | 3.29 | (2.47 , 4.12) | <.001 | 2.29 | (1.43 , 3.16) | 0.06 |
| Behavioral Disorder | 0.77 | (0.41 , 1.13) | 0.15 | 1.37 | (0.86 , 1.88) | 0.23 | 1.36 | (1.00 , 1.71) | 0.09 |

*Note.* Estimates are adjusted for age, sex, race, and insurance type. P-values calculated using Wald 2-tailed z-test. RRR=relative risk ratio.
